# Navigating the Challenges: A Commentary on Barriers to Autism Screening in Childcare Centers

**DOI:** 10.3390/bs16010079

**Published:** 2026-01-06

**Authors:** Andrea Trubanova Wieckowski, Georgina Perez Liz, Elizabeth McGhee Hassrick, Emmanuel Koku, Erika Frick, Autumn Austin, Diana L. Robins

**Affiliations:** 1 A.J. Drexel Autism Institute, Drexel University, Philadelphia, PA 19104, USA; gmp69@drexel.edu (G.P.L.); emh347@drexel.edu (E.M.H.); ef563@drexel.edu (E.F.); aa4642@drexel.edu (A.A.); dlr76@drexel.edu (D.L.R.); 2 Department of Sociology, Drexel University, Philadelphia, PA 19104, USA; efk26@drexel.edu

**Keywords:** early detection, autism, screening, childcare centers

## Abstract

Although the American Academy of Pediatrics has long recommended universal autism-specific screening at well-child pediatric visits, implementation challenges in primary care settings interfere with high-fidelity universal autism screening. These challenges delay autism identification for some children, leading to delays in needed services and supports. Prior findings indicate that new solutions must be developed to bridge the gap in access to autism screening for families, particularly among those who are under-resourced. One approach is expanding screening to other community settings, such as childcare centers, but there are barriers to this approach, which this commentary aims to address. We discuss challenges and barriers in childcare screening identified through our recently completed pilot study screening for autism in childcare centers, with suggested strategies to address them. These challenges include hesitation among childcare staff to guide conversations or concerns about autism, and stigma around autism diagnosis and presentation. Other challenges relate to emerging concerns regarding legal, ethical, and professional roles and responsibilities surrounding informed consent and data privacy, as well as the identification of children without timely follow-up evaluation and services. There is a need for increasing public awareness as an essential component of autism screening across settings. Our commentary discusses different considerations and practice strategies to meet these needs.

## 1. Lay Summary

Early identification of autism is critical to support learning, development, and positive outcomes for autistic children. One strategy to support early identification is to use screening tools during pediatric well-visits. However, many children—especially in under-resourced communities—face delays in early identification due to barriers they experience in primary care, for instance, during well-child check ups. This paper discusses the potential of expanding screening to other community settings, namely childcare centers, to help offset the barriers to early identification. We highlight challenges of childcare screening, such as staff discomfort, stigma, and privacy concerns, and offer strategies to address these issues. Increasing public awareness is emphasized as a key step in improving early identification of autism.

## 2. Navigating the Challenges: A Commentary on Barriers to Autism Screening in Childcare Centers

Autism Spectrum Disorder (herein referred to as autism) represents a significant public health concern. With prevalence currently estimated at over 3% of 8-year-old children in the United States (1 in 31; Shaw et al., 2025) and 1% estimated globally (Zeidan et al., 2022), it is imperative to explore strategies to increase autism screening and detection for all children. Early intervention for autism is crucial for improved outcomes in cognitive abilities, language abilities, educational outcomes, and social skills for children diagnosed with autism (e.g., Clark et al., 2018; Dimian et al., 2020; Vivanti et al., 2016). Further, there is evidence that autistic children benefit more from autism-specific intervention than general early intervention services such as speech, occupational therapy, or special instruction (Reichow et al., 2018). Evidence-based autism-specific interventions like Naturalistic Developmental Behavioral Interventions (NDBIs) are often more intensive (i.e., provided for more hours than other early interventions), target the core symptoms of autism using specific curricula to support the development of social engagement and social communication, and require specific training to deliver with high-fidelity, contributing to the benefits. However, before they are officially diagnosed with autism, children rarely access intensive, specialized autism intervention (Lord et al., 2012; Tomlin et al., 2013), leading to lifelong disparities compared to children who start autism-specific interventions at younger ages. The median age of autism diagnosis in the United States continues to be near the fourth birthday (Shaw et al., 2025), with global estimates of 48–60 months (van’t Hof et al., 2021), even though caregivers of autistic children are often concerned about their child’s development before the age of two (Guinchat et al., 2012; L. Herlihy et al., 2015). These delays in diagnoses directly lead to later onset of autism specific interventions, highlighting the need for early detection.

The American Academy of Pediatrics recommends autism-specific screening for all children at 18- and 24-month well-child visits, together with ongoing surveillance at all well-child visits and developmental screening at 9, 18, and 24/30 months (Hyman et al., 2020). It has been shown that universal early autism-specific screening can decrease the average age of diagnosis (Robins et al., 2014), especially for children with more subtle clinical presentations (Vivanti et al., 2025) and reduce racial/ethnic and socioeconomical disparities in autism detection (L. E. Herlihy et al., 2014). However, implementation challenges in primary care settings interfere with high-fidelity universal autism screening. Specifically, referrals for evaluation and intervention for children who screen at higher likelihood of autism remain low (Downs et al., 2019; Monteiro et al., 2019). Additionally, patients from minority groups in the United States are screened less often (Carbone et al., 2020; Guthrie et al., 2019; Zuckerman et al., 2013) and those from low-income households may be less likely to regularly attend checkups (Liptak et al., 2008; Wolf et al., 2018, 2021), resulting in later referrals for evaluation and services. These findings indicate that new approaches must be developed to bridge the gap in autism screening for families, particularly those who are under-resourced.

One approach is to consider alternative community settings outside the medical environments in which universal screening is recommended. In the United States, childcare centers provide care for up to 38% of all children under the age of 5 (U.S. Census Bureau, 2023), including close to 1 million children from underserved backgrounds who receive Head Start benefits for subsidized or free childcare (Office of Head Start, 2025). Thus, childcare centers may be an alternative setting for autism screening (Branson et al., 2008), especially for children who are most vulnerable to being diagnosed late because of cultural or socioeconomic factors, or limited access to healthcare. Autism-specific screening usually relies on caregiver report; childcare centers could serve as alternate venues to reach caregivers. Furthermore, expanding screening beyond caregiver reports and obtaining teacher input may identify behaviors that may manifest differently across settings (De Los Reyes et al., 2013).

Childcare teachers may provide a helpful perspective about children’s behavior and development, given that they regularly observe children’s play (Branson et al., 2008; Mitchell & Locke, 2015), are familiar with the usual behavior of children in their group, can observe children’s interactions with peers, and have knowledge and training in child development. Developmental screening, such as Ages & Stages Questionnaire (ASQ-3; Squires et al., 2009), is recommended for use in childcare by several agencies, including the Centers for Disease Control and Prevention (2024), Early Childhood Technical Assistance Center (2025), Zero to Three (2025), and Head Start (2024–2025). It is also a common practice in many certified childcare centers to assess for key developmental milestones in areas such as language, movement, and behavior. However, there are no such recommendations for autism-specific screening in childcare centers, resulting in absence of routine screening, and lack of information on acceptability of this practice in childcare centers. While general developmental screening aids in identifying children with any developmental delays including autism, autism-specific screening identifies some children with autism who screened negative on a developmental screener (Hardy et al., 2015; Wiggins et al., 2012).

Several studies have examined universal autism screening in childcare centers, which include childcare centers and preschools, with the goal of generating knowledge about feasibility, acceptability, effectiveness, and identifying barriers and challenges to implementation. A recent systematic review (DeLucia et al., 2024) found evidence of reliability and validity of autism screening by childcare providers, though only 5 of the 31 studies focused specifically on childcare settings, whereas other studies focused on older children in preschool, kindergarten, or across settings. In a pair of studies in Belgium (Dereu et al., 2010, 2012), teachers used the Checklist for Early Signs of Developmental Disorders, a screener specifically designed for childcare workers and healthcare providers to screen for autism in childcare centers and found several barriers to screening. These included a high false positive rate and low caregiver acceptability for questionnaires and clinical evaluation. Importantly to the question of feasibility of screening, teachers completed the questionnaires first, and caregivers needed to opt-out (i.e., decline participation) if they did not want to participate. Therefore, inclusion of teacher data was not dependent on caregiver action to agree to participate. In another pair of studies conducted in Norway, Larsen et al. (2018a, 2018b) developed a short observation list to be used by childcare teachers. Results indicate high agreement between caregivers and teachers (Larsen et al., 2018a), but insufficient properties (i.e., high false positive rate) to be independently used in childcare centers to identify autism (Larsen et al., 2020).

In contrast, a US study (Janvier et al., 2016) showed more promising results regarding screening for autism in underserved communities. Acceptability for screening was high (88%), and early childcare providers completed screeners for 90% of the children for whom caregivers gave consent. However, although 88% of caregivers consented to screening, which may reflect setting (80% were from Head Start sites) and age (3 to 5 years), many caregivers who were offered an autism evaluation for their child declined, demonstrating low acceptability of the follow-through actions when a child screens positive. These prior studies indicate a gap in knowledge regarding acceptability and feasibility of autism screening in childcare centers serving toddlers. Uzonyi et al. (2022) conducted interviews at childcare centers to understand perceptions on screening in childcare settings and found that most directors and teachers were “cautiously open” to autism screening but wanted more training, and most were open to universal autism screening at their child’s center. Together, these studies indicate preliminary acceptability and feasibility of screening in childcare centers for early detection of autism. However, many used novel measures, and no prior studies have explored the implementation and accuracy of the most commonly used autism screener for autism, the Modified Checklist for Autism in Toddlers, Revised, with Follow-Up (M-CHAT-R/F; Robins et al., 2014), in childcare centers, leaving a gap regarding its utility in this setting across informants.

In a recently completed project (Frick et al., 2024) we aimed to implement autism screening in childcare settings using the M-CHAT-R/F alongside developmental screening, and to compare teacher and caregiver report of the child’s usual behavior. The study was approved by the university Institutional Review Board, and consisted of two phases: first, to collect qualitative data regarding the barriers and facilitators of screening in childcare to inform a pilot study on effectiveness of implementing screening in a community setting; and second, to implement screening through caregiver and teacher report, using strategies informed by the findings in first phase. We partnered with local organizations that work directly with childcare centers to build trust and relationships, and enhance recruitment. In the qualitative data part of the study, we conducted interviews with caregivers (n = 9), childcare teachers (n = 8), and directors (n = 5), as well as conducted observations (n = 15) across 10 centers.

From the interviews and observations, a recommendations memo was created to inform strategies for successful screening implementation. These strategies included (1) Teacher and staff training, (2) Caregiver training, and (3) Childcare center implementation strategies. Our study offered a brief training for teachers and directors, utilizing both in person and virtual modalities, limited to 1 h to minimize burden. Trainings included background information about autism such as what autism is, how it is diagnosed, prevalence rates, and early signs and symptoms, as well as information about screening tools, and study logistics. The training was interactive, including discussion of the autism signs observed in video examples and opportunities to talk about experiences working with children with possible autism. Training also reviewed steps for teachers to take after screening is complete, including tips for discussing concerns with the caregivers. We partnered with First Up, an early childhood education organization that provides training for teachers and families. First Up staff reviewed and contributed to the training material, co-presented trainings with us, and offered the teachers state Quality Assurance System credits (akin to continuing education) as compensation. We also offered animated short videos for caregivers describing autism and the study. To reach as many families as possible, we offered both online and paper screening, we set up a table at centers with recruitment materials, attended family events such as back-to-school nights, and engaged in outreach through emails, apps used by childcare centers to communicate with caregivers, and outreach from the directors of centers, given their established relationship with their families. Due to recruitment challenges, we reduced the burden of participation for both teachers and caregivers by making developmental screening (ASQ-3 and ASQ: Social Emotional [ASQ:SE-2]; Squires et al., 2015) optional, reducing the time for participation to less than 10 min to complete consent, demographics, and M-CHAT-R/F.

Even though all directors were very responsive and enthusiastic about the study, after implementing all of the strategies mentioned above, only 10 out of 34 (29%) enrolled centers participated in the qualitative phase of the study (interviews and observations), and only three childcare centers carried out the quantitative phase (autism screening plus optional developmental screening). From those three centers that participated in the quantitative phase, only 15 caregivers (7%) completed screening for their children, out of 218 eligible children. Among the 15 enrolled children, we were successful in obtaining 14 completed teacher forms (93%), indicating that once a family consented, it was feasible to also collect the teacher screening. Of note, screening results were only shared with the caregivers, not the teachers. This was one of the strategies to assure caregivers that their privacy was intact, even though we were soliciting input from their child’s teacher. Children who were screened were classified into two groups, according to the scores obtained on all caregiver or teacher completed screeners (M-CHAT-R/F, ASQ-3, or ASQ:SE-2): low likelihood (continue usual monitoring of development recommended; n = 8), or high likelihood/moderate likelihood/monitor for autism (diagnostic evaluation recommended; n = 7) based on one or more scores from caregiver and/or teacher (see Table 1).

The agreement between caregivers and teachers was high for M-CHAT-R/F: 13 caregivers and teachers had congruent screener results, 1 dyad had incongruent screening result, and 1 teacher report was missing. The agreement between teachers and caregivers was more variable on the ASQ-3 and ASQ:SE-2, although only 5 teachers completed those screeners. Four of the 6 children in the elevated likelihood for autism (diagnostic evaluation recommended) category attended an evaluation; two children were diagnosed with autism, one with unspecified communication disorder, and one did not meet criteria for any diagnosis (see Table 1). The caregivers of other two children were non-responsive to calls to schedule the evaluation.

Findings from the pilot study demonstrate low acceptability of autism screening in childcare settings, given that investigators were not successful in enrolling caregivers in the quantitative screening component, despite incorporating feedback from caregivers, teachers, and directors during the qualitative component of the study to inform implementation. From those that did screen however, the proportionally high screen positive rate from caregivers suggests that caregivers may have been motivated to participate if they had developmental concerns about their child. We were unsuccessful engaging families without concerns, even with content in the caregiver videos about the importance of universal screening, rather than only screening when someone has concerns for a child’s development. This indicates selection bias in caregiver participation, which limits the generalization of the pilot study’s findings to universal autism screening in childcare centers. Of note, however, the high teacher completion rate demonstrates that once caregivers enrolled, they were comfortable authorizing teachers to complete screening. It also speaks to the feasibility and acceptability of teacher screening, confirming that they had capacity to complete the screening.

Admittedly, the low numbers of children that were evaluated during the pilot screening study are not robust enough to inform the accuracy of screening in childcare centers, and further research is needed after addressing barriers discussed below. Our results do speak to the potential for screening in childcare settings to identify children with possible autism, since two children without a previous autism diagnosis were identified through this study. One child was identified by both caregiver and teacher screening, whereas the other child was only identified through a caregiver report.

### Challenges/Barriers and Suggested Strategies to Address Them

In our study, the percentage of screening completion was very low even after reducing participation burden to less than 10 min. Since this was a research study, caregivers and teachers were asked to sign an informed consent to participate, which could have provided an additional barrier that would not be expected in a non-research context. However, the childcare centers reported that it is not unusual for them to have incomplete developmental screening forms (ASQ-3) from caregivers as part of routine practices; in fact, some centers indicated they do not even ask caregivers to complete it anymore, given history of low completion. Furthermore, autism screening is currently not part of the usual practices in childcare centers; our Institutional Review Board required active consent from caregivers before we asked teachers to complete screening forms, rather than a “opt-out” approach that was used in prior literature (e.g., Dereu et al., 2010). Despite the extensive collaboration with childcare centers, the high level of engagement from teachers and directors who attended our training, lay-friendly audiovisual resources aimed at engaging families, and appearance of study staff at childcare center events, participation was low, indicating low acceptability of autism screening in childcare centers.

We identified several barriers to screening from the qualitative arm of the study (Frick et al., 2024). First, teachers shared that they avoided conversations related to questions or concerns about developmental delays or autism with caregivers to avoid negative attribution surrounding autism. Instead, they sought out guidance and support from childcare directors, who counseled a “wait and watch” approach to discussions about autism. Teacher hesitation to approach caregivers directly with concerns may constitute a professional boundary in itself. The National Association for the Education of Young Children (NAEYC) offers “giving advice beyond the scope of one’s role or expertise” as an example of professional boundary crossing (NAEYC, 2020). The way that childcare professionals define their role and its limits might conflict with the implementation of practices that may be perceived as predominantly belonging to the medical field. Strategies to address these barriers might include fostering interdisciplinary collaboration and conversation to underscore that non-clinical staff are not expected to make medical decisions or diagnoses, but are equipped to effectively participate in the screening process within their professional scope, using screening results to engage in discussions about children’s behavior. Another strategy is specifically including content on autism identification and how to conduct these conversations with caregivers as part of the professional development curricula for childcare teachers, to increase their knowledge and confidence on the topic.

Second, teachers shared reservations about bringing up specific developmental concerns, specifically autism, due to stigma around the diagnosis and to avoid possible conflict with caregivers. To address this barrier, community-based participation approaches will be crucial to engage caregivers, childcare teachers and directors, and community leaders in contributing to the design and communication strategies of screening programs. Connecting caregivers who champion early detection and intervention based on their lived experiences with skeptical caregivers could potentially help overcome barriers related to distrust. Providing screenings that are supportive and empowering, rather than labeling or alienating, will also be central to engagement efforts. In addition, expanding autism screening to other community settings such as places of worship (i.e., churches, synagogues or mosques) or community centers may increase community support and awareness. Community-based Organizations (CBOs) who are trusted by community members could be trained and empowered to lead autism screening education in order to increase its acceptability and cultural resonance with their specific community. Childcare providers can model for families how to address questions about autism in an accepting and affirming way that could, in turn, contribute to reducing stigma.

Third, concerns about legal, ethical, and professional roles and responsibilities regarding informed consent and data privacy are another major consideration. Since autism screening in childcare is not currently routine practice, caregivers needed to consent and complete the screener before the teachers were asked to participate. The low rate of engagement from caregivers limited the ability to test the efficacy of autism screening in childcare. The low response rate precluded the investigators from examining the concordance in screening scores between different informants (i.e., caregivers and teachers).

In some settings, screening programs have been criticized for identifying children with high likelihood of developmental delays without being able to ensure timely follow-up evaluation and services (e.g., King et al., 2010). This concern can lead to low acceptability from teachers and families. This issue is well-documented in the context of autism and other developmental conditions, where children who screen positive may face long wait times, geographic inaccessibility, and shortages of specialists trained in early childhood diagnostics. For example, research has shown that after a positive autism screen, families—especially those from low-income or rural communities—often experience significant delays in accessing follow-up assessments and services (Karp et al., 2018; Zuckerman et al., 2015). Although autism specific interventions can have benefits at any age, studies show the greatest benefit when intervention is implemented early (e.g., Dawson et al., 2010; Guthrie et al., 2023; Vivanti et al., 2016). Therefore, delays in access to diagnostic and intervention services have serious repercussions. Moreover, families may become discouraged or disengaged during long referral processes, contributing to lower follow-through rates (Carbone et al., 2010).

Beyond recognizing practical and logistical barriers to screening in our study, the challenges associated with implementing screening in childcare centers highlight critical considerations to public health. Primary prevention strategies, which aim to avoid the onset of a disease or condition are well established within educational settings (e.g., vaccination requirements). This is likely because primary prevention in early childhood prioritizes decreasing exposure to infectious diseases. In contrast, secondary prevention strategies, which aim to identify a condition as early as possible to intervene early and improve outcomes, remain underutilized in childcare settings—particularly for developmental delays and autism.

Increasing public awareness about developmental and autism screening as essential components of secondary prevention is imperative and should be prioritized accordingly. In the healthcare field, some secondary prevention strategies are implemented universally (to all children of a certain age, such as the neonatal screening panel for congenital metabolic disorders) in contrast to others implemented selectively, or only to children with known factors that increase likelihood of the condition, like screening for hip dysplasia in newborns who were breech (Campbell et al., 2014). Some of the universal screening strategies (such as hearing or vision screening) in early childhood may be familiar to caregivers, and might serve as examples to relate to. If caregivers and teachers relied only on their anecdotal non-standardized observations about a child’s vision or hearing to determine whether or not the child requires formal diagnostic determination, they would identify some cases but would likely miss less obvious ones or identify them much later. However, universal screening greatly reduces the probability of missing a child who might benefit from a diagnostic evaluation (Wang et al., 2011).

When addressing the importance of screening, it is also essential to convey that a positive screen does not equate to a diagnosis but rather identifies the need for further clinical evaluation to determine if a diagnosis or educational classification is appropriate, and more importantly, to identify supports and services that could benefit the child and family. While there are no screening tools that are perfectly infallible, standardized screeners inform users about how to interpret a positive screen and provide guidance on the next steps for the child, including recommendations for diagnostic evaluations and/or intervention and other family supports.

Furthermore, greater dissemination of information like the Centers for Disease Control and Prevention (CDC)’s “Learn the Signs. Act Early” campaign (Centers for Disease Control and Prevention, 2025), accessible both to caregivers and teachers, is needed to help normalize the idea of monitoring child development using a more comprehensive approach, and moving beyond a focus on commonly recognized milestones, such as crawling, first words, or first steps. Motor and language milestones may be considered more objective, whereas social and emotional development milestones are more subjective and report of these can be influenced by the observer’s social references of “adequate” behavior and sometimes confounded with parenting styles. As such, delay in acquiring motor or language development milestones might carry less stigma than socio-emotional development delay. Emphasizing the significance of social-emotional development milestones is crucial for elevating the priority of autism screening within the educational environment.

**Figure 1:**
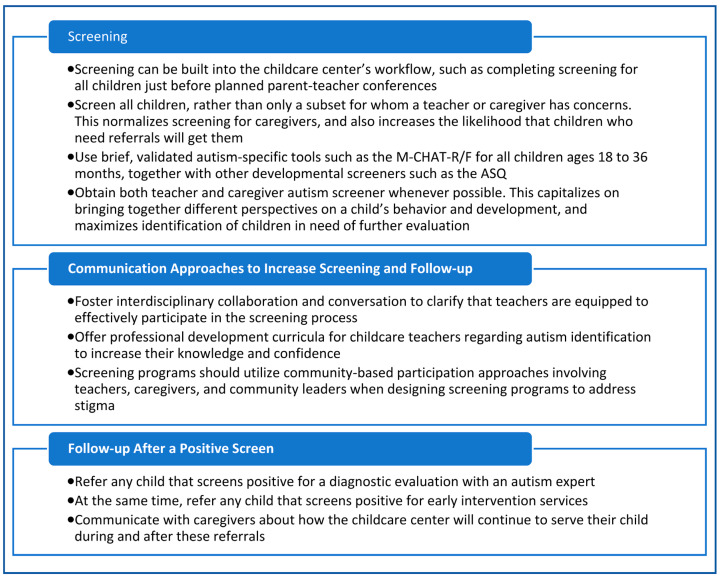
Best practices for autism screening in childcare centers.

Of note, a limitation of this commentary is that the challenges and suggested strategies around autism screening are discussed within the United States framework, limiting application outside of the United States where childcare systems as well as screening recommendations differ. In addition, the preliminary data used to illustrate the challenges was collected as part of a research study, limiting our understanding of screening in childcare centers as part of routine care. The ideas presented within this commentary, however, serve to highlight the challenges and discuss some potential strategies for addressing the challenges. Figure 1 highlights best practices for autism screening in childcare centers.

Overall, the aim of this commentary is to further the conversation regarding the implementation of autism screening outside of healthcare settings, particularly in childcare centers. Given the barriers to screening during pediatric well-child visits, it is important to evaluate whether other professionals who often interact with young children, such as teachers, can utilize existing screening tools to identify children at higher likelihood of autism. We discuss several barriers to screening in childcare centers, and offer strategies to address them. These barriers include teacher avoidance to bring up concerns regarding developmental delays or autism with caregivers, stigma around the diagnosis, concerns regarding legal, ethical, and professional roles and responsibilities regarding informed consent and data privacy, as well as ethical implications of screening without timely follow-up evaluation and services. Lastly, we discuss the need for increasing public awareness about developmental and autism screening as public health measures to aid in early identification. It is crucial to continue investigating specific barriers and strategizing to address them effectively. An important consideration is that challenges and barriers to identifying children who require an autism diagnostic assessment and services may not be solely defined by the nature of the childcare provider role. Rather, the dynamics between caregivers and teachers to address caregiver concerns or conduct routine screening are likely shaped by other contextual factors, such as the structure of health and educational systems in different regions of the world, and even the culture and values of different populations. It will be beneficial to young children and their families to increase awareness and self-efficacy in childcare teachers regarding autism early detection, and to raise the visibility of social-emotional development as part of the integral child health and wellbeing.

## Figures and Tables

**Table 1:** Participant information and screener results for pilot screening in childcare centers.

Child	Sex	Age (Months)	Positive Screener(s)	Outcome
1	F	29	Teacher: ASQ:SE-2	Evaluated (TD)
2	M	39	Caregiver: ASQ:SE-2	Lost to follow-up
3	M	16	Teacher: ASQ-3	Lost to follow-up
4	F	15	Caregiver: ASQ-3 Teacher: ASQ-3	Lost to follow-up
5	F	27	--	No evaluation needed
6	F	38	--	No evaluation needed
7	M	26	--	No evaluation needed
8	M	35	Teacher: ASQ-3	Evaluated (Unspecified Communication Disorder)
9	M	36	Caregiver: M-CHAT-R/F, ASQ:SE-2, ASQ-3 Teacher: M-CHAT-R/F, ASQ:SE-2, ASQ-3	Evaluated (ASD)
10	F	31	--	No evaluation needed
11	F	17	--	No evaluation needed
12	F	41	--	No evaluation needed
13	F	34	Caregiver: M-CHAT-R/F, ASQ:SE-2, ASQ-3	Evaluated (ASD)
14	M	22	--	No evaluation needed
15	F	22	--	No evaluation needed

Note. M-CHAT-R/F—Modified Checklist for Autism in Toddlers, Revised, with Follow-up. If the initial score is <3 = negative; If the initial score is 3–7, administer M-CHAT Follow-Up, if M-CHAT Follow-Up score is +2 = Positive; If the initial score is > or = to 8, Positive. ASQ:SE-2—Ages and Stages, Social Emotional, 2nd Edition. If child fell into the monitor or positive area, specific to their age, they were invited for an evaluation. ASQ-3—Ages and Stages, 3rd Edition. If child fell into the monitor or refer area for the Communication domain OR the Personal-Social domain, they were invited for an evaluation. Screeners not listed were either missing or were negative. Outcome—“No evaluation needed”—all screeners were negative; “Lost to follow-up”—at least one screener was positive, but the family did not attend the evaluation to determine final outcome; “Evaluated”—child was evaluated and diagnosed with “ASD”—Autism Spectrum Disorder, “Unspecified Communication Disorder” or the child was evaluated, but did not meet the criteria for any mental health diagnosis “TD”. Missing data can be attributed to a change in study requirement due to low enrollment, so requirement shifted to M-CHAT completion, with ASQ-3 and ASQ:SE-2 being optional.

## Data Availability

The data presented in this study are openly available in NIMH Data Archive at DOI: 10.15154/2h10-1053.

## Abbreviations

ASQ-3	Ages & Stages Questionnaire
ASQ:SE:2	Ages & Stages Questionnaire: Social Emotional
CBO	Community-based Organizations
CDC	Centers for Disease Control and Prevention
M-CHAT-R/F	Modified Checklist for Autism in Toddlers, Revised, with Follow-Up
NDBI	Naturalistic Developmental Behavioral Interventions
NAEYC	National Association for the Education of Young Children
US	United States
